# The Significance of IL-36 Hyperactivation and IL-36R Targeting in Psoriasis

**DOI:** 10.3390/ijms20133318

**Published:** 2019-07-05

**Authors:** Stefania Madonna, Giampiero Girolomoni, Charles A. Dinarello, Cristina Albanesi

**Affiliations:** 1 Laboratory of Experimental Immunology, IDI-IRCCS, via Monti di Creta, 104, 00167 Rome, Italy; 2 Section of Dermatology, Department of Medicine, University of Verona, P.zza Stefani, 1, 37126 Verona, Italy; 3 Department of Medicine, Radboud University Medical Center, 6525 HP Nijmegen, The Netherlands; 4 Department of Medicine, School of Medicine, University of Colorado, Denver, Anschutz Campus, Aurora, CO 80045, USA

**Keywords:** psoriasis, IL-36, IL-38, IL-17, skin inflammation

## Abstract

Psoriasis is an immune-mediated inflammatory skin disease that involves mainly T helper (Th)17, Th1 and Th22 lymphocytes, which cause hyper-proliferation of the epidermis with aberrant differentiation of keratinocytes, and local production of chemokines and cytokines. These fuel a self-amplifying loop where these products act on T cells to perpetuate cutaneous inflammatory processes. Among the various inflammatory mediators involved, interleukin (IL)-36 cytokines are important for the recruitment and activation of neutrophils and Th17 cells in psoriatic skin. In particular, IL-36s induce chemokines and cytokines interfere with differentiation/cornification programs in the epidermis, as well as promote pathological angiogenesis and endothelial cell activation. IL-36 cytokines belong to the IL-1 family, and comprise IL-36α, IL-36β, and IL-36γ agonists as well as IL-36 receptor antagonist and IL-38 antagonists. IL-36 cytokines are up-regulated in psoriatic epidermis, and their expression is strongly induced by TNF-α and IL-17. Contrarily, IL-38 antagonist is downregulated, and its impaired expression may be relevant to the dysregulated inflammatory processes induced by IL-36. Here, we discuss on the pathogenic mechanisms leading to the altered balance of IL-36 agonists/antagonists and the significance of this dysregulation in psoriasis. Collection of the information will provide a theoretical basis for the development of novel therapeutic strategies based on IL-36 agonist/antagonist manipulation in psoriasis.

## 1. Introduction

**Figure 1:**
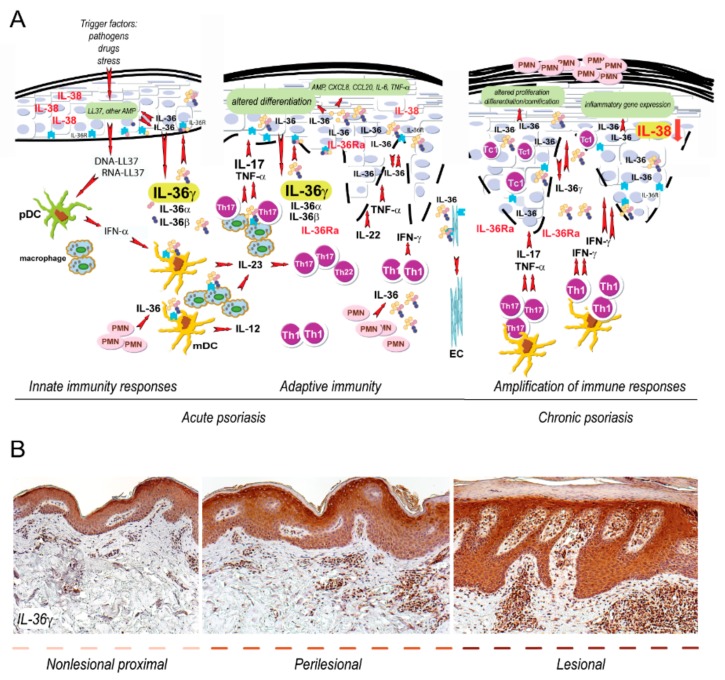
IL-36 circuits are hyperactivated in both acute and chronic phase of psoriasis. (**A**) Early events in psoriasis include induction of innate immunity pathways. During this phase, keratinocytes produce antimicrobial peptides, in particular the cathelecidin LL37. The latter activates pDC and mDC, which trigger, via IFN-α and IL-23/IL12, the T helper (Th)17/Th22 and Th1 responses, thus initiating the adaptive immune phase. In both acute and chronic psoriasis, the expression of IL-36 cytokines is strongly upregulated by stimuli typical of innate or adaptive immunity, respectively. IL-36γ, for instance, can be induced, together with the other IL-36 family members and IL-36Ra, by LL37 and TNF-α, as well as by IL-17 and IL-22. Contrarily, inflammatory cytokines down-regulate the expression of IL-38 in skin lesions. IL-36s activate pathogenic pathways in different cell types in psoriatic skin, especially in resident skin cells, such as keratinocytes and endothelial cells (EC). In keratinocytes, IL-36s promote the release of inflammatory molecules and interferes with differentiation/cornification processes of the epidermis. IL-36s also regulates the production of IL-23 and IL-12 by mDC and macrophages, thus influencing the driving of Th17 and Th1 responses in psoriatic skin. IL-36 cytokines also sustain the recruitment of neutrophils (PMN) in psoriatic skin. T-cell infiltrate present during late/chronic phase of psoriasis establishes a cytokine milieu, that further upregulate the keratinocyte production of IL-36. (**B**) Both acute and chronic phases of psoriasis can be found within the same psoriatic plaque, being it comprehensive of nonlesional proximal to perilesional and lesional areas, with IL-36γ expression predominantly present in the epidermis of lesional skin.

Psoriasis is a complex skin disease with autoimmune components that involves resident skin cells and immune cells, generating self-sustaining inflammatory cycles. Psoriatic lesions manifest as hyperkeratotic plaques and are caused by the infiltration of effector leukocytes which induce hyperproliferation of the epidermis with aberrant differentiation of keratinocytes, dermo-epidermal inflammation, and angiogenesis [1,2]. Early upstream events occurring in psoriasis include the induction of innate immunity pathways and responses in keratinocytes [3] (Figure 1). In this phase, keratinocytes overproduce antimicrobial peptides (AMP), cytokines of IL-1 family, and chemoattractants, altogether important for the activation and recruitment of innate immunity cells such as plasmacytoid dendritic cells (pDC), neutrophils, and macrophages [4,5]. Among keratinocyte-derived AMP, the catheledicin LL37 has a pivotal role in the development of psoriasis, through its capacity to activate pDC and myeloid DC (mDC), with consequent initiation of the adaptive immune phase [6,7] and activation of the IL-23–T helper (Th)17/Th22 and IL-12-IFN-γ/TNF-α axes [2]. While IL-17/IL-22-producing T cells are mainly found in the initial phases of psoriasis, IFN-γ-producing Th1 and Tc1 cells accumulate during the chronic phase of the disease. Hence, T-cell infiltrate in active psoriatic skin establishes a cytokine milieu that concurs to derange proliferative and differentiation programs of the epidermis, and induce the local overexpression of several mediators of inflammation [8,9]. An unceasing self-amplifying loop sustained by keratinocytes and adaptive immunity cells further intensifies inflammation and leads to disease perpetuation and maintenance (Figure 1). Among the variety of pathogenic mechanisms operating in psoriasis, those mediated by IL-36 cytokines have been put forward as very relevant [10,11,12]. IL-36 cytokines are a subgroup of the wider IL-1 family, comprising three agonists, IL-36α, IL-36β, and IL-36γ, and two antagonists, IL-36 receptor antagonist (known as IL-36Ra or IL-36RN) and IL-38, with opposite functions and competitively binding to IL-36R/IL-1R/AcP complex. While IL-36α, IL-36β, and their antagonists, in particular IL-38, are physiologically present in healthy skin, with a prominent localization in the epidermal compartment, IL-36γ can be significantly detected only in psoriatic lesions. Here, IL-36 cytokines, in particular IL-36α, IL-36γ and IL-36Ra, are highly produced by epidermal keratinocytes, and at lower extent, by dermal fibroblasts and endothelial cells (Table 1). Concomitantly, IL-38 expression is downregulated in the epidermis of psoriatic lesions [13] (Table 1). IL-36 cytokines, mostly IL-36α and γ, are also produced by macrophages, Langerhans cells, and dendritic cells infiltrating dermis of psoriatic skin [14] (Table 1). 

The significance of IL-36 in psoriatic inflammation has been [15,16,17]. IL-36 cytokine upregulation in psoriasis occurs both in the early and in the late phases of the disease (Figure 1). In both phases, IL-36 expression is induced and sustained by stimuli typical of innate or adaptive immunity, characterizing acute and chronic skin lesions, respectively. IL-36γ, together with other IL-36 family members and IL-36Ra, can be, in fact, strongly induced by LL37, TNF-α, and IL-36 themselves, as well as by IL-17 and IL-22, which accumulate in acute skin lesions [18]. Contrarily, the expression of IL-38 antagonist is down-regulated by inflammatory cytokines, and in particular by IFN-γ, abundantly present in chronic plaques. Therefore, the unbalanced expression levels of IL-36/IL-38 may be responsible for the dysregulated inflammatory processes induced by IL-36 in psoriatic skin, especially in the epidermal compartment [13]. 

IL-36R-mediated signal transduction has been shown to induce a number of pathogenic pathways in different cell types of psoriatic skin, especially in resident skin cells such as keratinocytes and endothelial cells (Figure 1). In keratinocytes, IL-36s promote the release of pro-inflammatory cytokines and chemokines, upregulate AMP and proliferative mediators, and, importantly, interferes with differentiation/cornification processes [19,20,21,22]. IL-36R-dependent signaling also induces the expression of T-cell attracting and adhesion molecules by endothelial cells [21], as well as regulates the production of T-cell polarizing cytokines by mDC and macrophages, thus influencing Th17 and Th1 responses in psoriatic skin [15,19,20,21,22,23]. Of note, IL-36 cytokines potently sustain neutrophil circuits in psoriatic skin, in particular in clinical variants of psoriasis where neutrophils are abundant and have a major pathogenic role, such as generalized pustular psoriasis (GPP) and palmoplantar pustolosis (PPP) [24]. Here, genetic mutations in IL-36 signature, and in particular in the *IL-36RN* gene, have been identified, confirming the pathogenic role of IL-36 axis in psoriasis development [25,26,27,28]. Interestingly, a higher frequency of *IL-36RN* disease alleles has been found in GPP compared to the PPP patient population, as well as associated with an earlier age of pustular psoriasis onset [29].

In this review, we discuss on the molecular and cellular mechanisms leading to the dysregulated IL-36 expression in psoriasis, with the emphasis on consequences of the altered balance of IL-36 agonists/antagonists in psoriatic skin. We illustrate the multiple pathogenic effects of IL-36 cytokines on inflammatory responses of resident skin cells and immune cells, as well as their impact on differentiation/cornification processes of the epidermis. Collection of the information will provide a theoretical basis for the development of new therapeutic strategies for psoriasis based on IL36R-targeting.

## 2. Structural and Functional Characterization of Il-36 Receptor

### 2.1. Organization of IL-36R Molecular Complex

Members of the IL-36 cytokine family were originally discovered due to their homology with two members of IL-1 family IL-1α and IL-1β. Following in vitro functional characterization and identification of their cognate receptor IL-36R, the IL-36 subfamily of cytokines was re-classified as three agonist cytokines IL-36α, IL-36β, and IL-36γ (previously termed IL-1F6, IL-1F8, and IL-1F9) and the natural antagonists IL-36Ra and IL-38, formerly known as IL-1F5 and IL-1F10, respectively [30].

IL-36 cytokines, such as all the IL-1 family members except for IL-1Ra, are synthesized without a signal peptide and therefore are not secreted via the endoplasmic reticulum-Golgi pathway and the mechanism of their release remains yet undefined [31]. Post-translational processing is required for full agonist or antagonist activity of all IL-36 members [5,32]. Similarly, recent in vitro and in vivo studies demonstrated that N-terminally truncated fragments of IL-38 (20–152 and 7–152 amino acid fragments) have increased biological activity compared to full-length form [33,34]. Neutrophil-derived proteases seem to be responsible for IL-36 agonists maturation [32,35]. However, it is unclear whether truncation of IL-36 agonists and antagonists occurs in humans under pathologic conditions.

**Figure 2:**
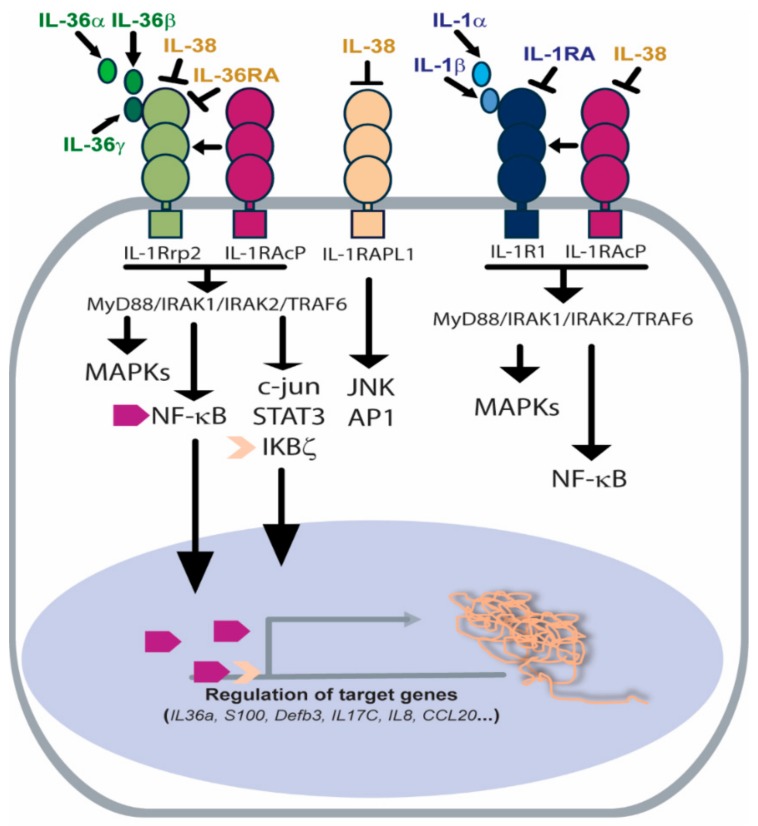
Receptor and signaling pathways activated by IL-36 agonists or inhibited by IL-36Ra and IL-38 antagonists. IL-36R heterodimeric complex is composed of IL-1Rrp2 subunit and IL-1RAcP1 co-receptor. IL-36 agonists bind IL-1Rrp2 and favour the recruitment of IL-1RAcP1, thus activating the MyD88/IRAK1/IRAK2/TRAF6 platform and the corresponding intracellular signalling, such as MAPKs, c-Jun, IKBζ/NF-κB and STAT3. IL-1RAcP1 co-receptor is shared by IL-1R complex, which, upon activation, leads to the activation of similar intracellular signaling. IL-36Ra and IL-38 antagonize with IL-36s for binding to IL-1Rrp2 and inhibit the corresponding cascades. Additionally, IL-38 binds the orphan IL-1RAPL1 receptor, blocking JNK and AP1 signaling.

Mechanistically, IL-36 agonists bind the same heterodimeric receptor complex, composed of IL-36R (initially termed IL-1R-related protein 2 also known as IL-1RL2 or IL-1Rrp2) and a co-receptor subunit IL-1 receptor accessory protein, IL-1RAcP1, which is shared by IL-1α, IL-1β and IL-33 [36] (Figure 2). IL-1Rrp2 contains a signaling peptide, an extracellular domain (ECD), a transmembrane helix and an intracellular Toll/IL-1 receptor (TIR) domain. The protein is localized to the plasma membrane as a single-pass transmembrane protein with the ECD on the cell surface and the TIR domain localized in the cytoplasm [36].

Although the crystal structure of IL-36γ has been resolved, the structure of IL-1Rrp2 or IL-1Rrp2/IL-1RAcP/ligand complex is not yet available. Yi et al. modelled the IL-36R ECD complexed to IL-36 cytokines and examined the structure and function of IL-36R using cell-based reporter assays [37]. The authors found that the presence of IL-36γ significantly increased the interaction between IL-1Rrp2 ECD and the accessory protein IL-1RAcP, leading to the activation of the intracellular NF-κB and MAPK signaling via MyD88/IRAK1/IRAK2/TRAF6 platform [37,38] (Figure 2). Interestingly, upon activation, the ECD domains of IL-1Rrp2 and/or IL-1RAcP, released extracellularly, could bind to co-receptor or IL-36 agonists, thus reducing the inflammatory responses in a regulatory feedback mechanism.

Although the organization and formation of IL-1R and IL-36R receptor complexes are quite similar, different mechanisms of intracellular trafficking involving Myd88/Tollip scaffold regulate their recycling and function. We can speculate that these differences in the trafficking and accumulation of the IL-36R and IL-1R could finely regulate the entity or timing of their corresponding intracellular signaling and contribute in a unique manner to the pathogenesis of inflammatory diseases [39].

### 2.2. IL-36R-Dependent Intracellular Signaling

IL-36R-dependent intracellular pathways has been widely investigated in human keratinocytes, which constitutively express high levels of IL-36Rrp2 and IL-1RAcP1 subunits and vividly respond to IL-36s stimulation (Table 1). Previous studies demonstrated in fact that IL-36α, β or γ agonists strongly activate NF-κB, and STAT3 pathways, thus inducing the expression of *IL-36α*, *S100a8*, *Defb3*, and *IL-17C* mRNAs in wild-type keratinocytes, but not in cells lacking of IL-36R. [40,41] (Figure 2).

Other than NF-κB signaling, IL-36γ also activates the phosphorylation of P38 kinase, known to participate to the induction of neutrophil chemokines IL-8, CXCL3 and CCL20 in human keratinocytes upon Toll-like receptor (TLR) activation [18,42]. Recently, the intracellular IκBζ mediator has been also uncovered as having an important role in the IL-36-induced intracellular signaling in epidermal keratinocytes. In particular, Muller et al. demonstrated that IL-36α and IL-36γ agonists are potent inducers of IκBζ expression, an atypical nuclear IκB protein, which acts not only as a repressor but, more importantly, as an activator of a selective subset of NF-κB-dependent inflammatory genes in human keratinocytes [43]. Endothelial cells also constitutively express IL-36R complex and respond to IL-36γ by activating the phosphorylation of NF-κB and c-Jun kinases, known to have pro-inflammatory functions [21] (Table 1) (Figure 2). Consistently, we recently observed that in these cells, IL-36γ strongly induces the phosphorylation of NF-κB/p65, but also of ERK1/2, which is known to induce proliferative responses in different cell types (unpublished data) (Figure 2). Human dermal fibroblasts also express active IL-36R complex and respond to IL-36 cytokines (as discussed in Section 5) (Table 1), although the corresponding intracellular signaling have not been investigated. Previous studies showed that the phosphorylation of P38 and HSP27 cascades is induced by IL-36γ in human synovial fibroblasts via activation of IL-36R complex [44]. In psoriatic skin, dendritic cells and macrophages efficiently respond to IL-36 stimulation in an IL-36R-dependent manner (Table 1), as discussed in Section 5, although a detailed characterization of the intracellular signaling is lacking.

Unlike IL-36 agonists, the binding of the two natural antagonists, IL-36Ra and IL-38, to IL-36R inhibits the activation of the corresponding intracellular pathways [21,44]. In vitro studies demonstrated that IL-36Ra binds to IL-36R much stronger than either IL-36α or IL-36γ and, in the presence of agonists, it competes with them for IL-36R occupancy [45] (Figure 2). Consequently, IL-36Ra binding to IL-36R impedes the recruitment of IL-1RacP for the ternary IL-36R: IL-1RAcP1:IL-36 complex formation, thus inhibiting the intracellular pathways, as clearly demonstrated in keratinocytes, endothelial cells, and synovial fibroblasts [18,21,44]. IL-38, the newest member of the IL-1 family, shares 41% homology with IL-1Ra, 43% homology with IL-36Ra and lower homology (14–30%) with IL-1β and other IL-1 subfamilies [46]. Similar to IL-36Ra, IL-38 binds to IL-1Rrp2 and inhibits the downstream cascades, even if it is unknown whether it blocks the recruitment of IL-1RacP to the receptor subunit [46]. We recently observed that, similarly to IL-36Ra, IL-38 impairs the phosphorylation of P65/NFκB and P38 in human keratinocytes induced by IL-36γ [18].

However, in addition to IL-36R, IL-38 can bind, albeit with low affinity, to IL-1R1, but also to the orphan receptor Three Immunoglobulin Domain-containing IL-1 receptor-related 2 (TIGIRR-2), formerly known as interleukin 1 receptor accessory protein- like 1 (IL-1RAPL1) [32] (Figure 2). Mora et al. recently demonstrated that IL-38 released by apoptotic cells inhibits the IL1RAPL1-dependent phosphorylation of JNK/AP1 cascade, thus limiting the production and release of inflammatory cytokines from human macrophages [33]. The ability of IL-38 to bind IL-1RAPL1 has been recently supported in vivo in the imiquimod (IMQ)-induced murine model of psoriasis. Here, a recombinant mature form of IL-38, but not IL-36Ra, is able to inhibit the IL1RAPL1 activation, thus suppressing the IL-17 production by dermal γδT cell [34]. Further studies would decipher whether, in addition to IL-1R1/IL1RAcP or IL-36R/IL-1RAcP complexes, IL-38 can bind other receptors from the IL-1 receptor family, such as IL-1R8 or IL-1R9, or other inflammatory receptors with a pathogenic role in psoriasis.

## 3. IL36R-Dependent Responses in Psoriatic Resident Skin Cells

### 3.1. Epidermal Keratinocytes

Several findings indicate that IL-36 is involved in skin inflammation, acting mainly on epidermal keratinocytes [47,48]. IL-36 agonists, as well as IL-36Ra, are released by keratinocytes upon stimulation with TLR agonists (lipopolysaccharide, poli I:C) or IL-1α, as well as by the inflammatory cytokines TNF-α, IL-17 and IL-22, which accumulate in the initial phases of disease. IL-36s are able to induce its own expression by acting in an autocrine loop [18,49]. Recently, it has been demonstrated that IL-36γ is also induced in epidermal keratinocytes by the neutrophil extracellular traps (NETs), large neutrophil-derived structures that contribute to the early psoriatic inflammation [50]. Contrarily, IL-38 expression is down-regulated by IL-22, IL-36γ and, interestingly, by Th1-released IFN-γ, thus contributing to the unbalance of the IL-36/IL-38 ratio also in the chronic skin lesions. In fact, although IL-36Ra antagonist is induced by inflammatory cytokines, its levels could be not sufficient to unleash the IL-36-dependent responses.

Other than releasing IL-36s, human keratinocytes vividly respond to them by producing several pro-inflammatory mediators, such as anti-microbial peptides, including AMP, LL-37, human β-defensin (HBD)-2, and S100 calcium-binding protein A7 (S100A7), the cytokines TNF-α and IL-6, and the neutrophil chemoattractant molecules CXCL8, CXCL1, CCL3, and CCL20, which act on immune cells, sustaining a self-amplifying inflammatory loop [18,20]. Furthermore, IL-36α and IL-36γ induce the expression and release of Heparin-binding EGF-like growth factor (HB-EGF) and, at lower extent, of vascular endothelia growth factor (VEGF)-A, two pro-proliferative factors acting on fibroblasts and endothelial cells, respectively [13,19]. Additionally, in human keratinocytes, IL-36γ, in synergy with TLR4 agonists, induces the release of lipocalin 2 (LCN2), further increasing neutrophil infiltration into the skin, and thereby amplifying the inflammatory cascade [50]. Interestingly, IL-36s strongly cooperate with IL-17A in inducing anti-microbial peptides, in particular HBD-2 and S100A7A, as well as pro-inflammatory cytokines, such as IL-6 and CXCL8, thus amplifying Th17 inflammatory responses [51].

Other than inducing inflammation, IL-36 cytokines, alone or in synergy with IL-17A, interfere with keratinocyte differentiation. In particular, N-terminally truncated forms of IL-36α, β and γ cytokines downregulate the expression of various epidermal differentiation markers, including filaggrin, involucrin, keratin 1 and keratin 10, in both keratinocyte cultures undergone terminal differentiation and skin equivalent models [13,52]. However, further studies are needed to delineate the molecular mechanisms triggered by IL-36s counteracting differentiation process in the skin. Interestingly, we demonstrated that IL-36R blockade by a recombinant full-length form of IL-38 restores keratinocyte differentiation impaired by IL-36γ, similarly to what was observed with IL-36Ra, by rescuing the physiological levels of differentiation markers [13]. Finally, exogenous IL-38 also suppresses the expression of pro-inflammatory molecules in human keratinocytes activated by IL-36γ, including IL-6, CXCL8, CCL20, and the antimicrobial peptides LL-37 and HBD-2 [13].

### 3.2. Dermal Endothelial Cells and Fibroblasts

In addition to human keratinocytes, dermal endothelial cells also express the IL-36R complex, and its activation by IL-36γ induces proliferation, as well as tube formation and branching [21,23] (Table 1). Consistently, we recently observed that supernatants from IL-17A-treated keratinocytes strongly promotes proliferation of dermal endothelial cell cultures, and this effect was specifically reduced by IL-36Ra treatment (unpublished data). IL-36γ, alone or in cooperation with TNF-α, also has pro-inflammatory effects on human endothelial cells, by inducing the release of IL-6, CXCL8, CCL20, CXCL1, and also expression of the adhesion molecules, such as the intracellular adhesion molecule (ICAM)-1 and vascular cell adhesion molecule (V-CAM), thus determining an increased migration of T cells [13,21,23]. These findings support a role for IL-36γ in enhancing psoriatic skin inflammation by activating angiogenesis and promoting leucocyte recruitment. In addition, IL-36γ induces proliferation and migration of dermal endothelial cells indirectly through the release of the pro-angiogenic molecule VEGF-A from dermal fibroblasts, being these last able to constitutively express high levels of IL-36R [23] (Table 1).

Interestingly, due to endothelial activation and increased monocyte adherence, this altered interplay between immune and resident skin cells evoked by IL-36R activation could explain the association with cardiovascular comorbidities occurring in psoriatic patients. As a consequence of IL-36R expression in endothelial cells, recombinant IL-38 has anti-inflammatory and anti-angiogenic effects on dermal endothelial cells activated by IL-36γ [13], whereas IL-38 action on dermal fibroblasts remains to be investigated. In these cells, IL1RAPL receptor, an aforementioned target of IL-38 cytokine, is constitutively expressed and strongly induced by psoriasis-related cytokines, including IL-17A, TNF-α, IFN-γ and IL-22 (our unpublished observation).

*Vice versa*, IL1RAPL1 expression is totally absent in human dermal endothelial cells and present, at low levels, in human keratinocytes. Further studies will be needed to delineate the function of this poorly characterized receptor in fibroblasts and the potential impact of IL-38 on its biological activity.

## 4. IL36R Function in Psoriasis Immune Cells

IL-36R function on inflammatory responses evoked by immune cells is supported by numerous studies [4,47]. Initially, a high expression of IL-36R mRNA levels was found in human Langerhans cells and dermal CD1a^+^ DC, together with CD14+ monocytes and CD11c+ mDC [19,53]. In these studies, the authors demonstrated that the immune cells strongly respond to IL-36β stimulation by upregulating surface expression of specific activation markers, such as CD83, CD86, and HLA-DR, and by inducing the production of inflammatory cytokines, such as IL-1β, IL-12, IL-23, IL-6 and TNF-α, but also chemokines, including CCL1, CXCL1 and GM-CSF in an IL-36R-dependent manner. Additionally, IL-36–matured DC strongly induced T cell proliferation [19,53] (Table 1).

Regarding T lymphocytes, in mouse IL-36R is primarily found on the surface of naive CD4^+^, but not CD8^+^, T cells, suggesting that IL-36s play a pivotal role in initiating the immune responses [54]. In these cells, IL-36s synergize with IL-12 to drive a potent Th1 response [55]. Unlike mouse cells, human CD4^+^ and CD8^+^ T cells do not express IL-36R nor respond to IL-36 either, when resting or under CD3/CD28 stimulation [19] (Table 1).

In addition to DC and T cells, IL-36 cytokines affect the function of macrophages, known to contribute to psoriasis pathogenesis [53]. An interesting characteristic of macrophages is their ability to switch from a pro-inflammatory M1 phenotype to an anti-inflammatory M2 phenotype, and thus participate sequentially in both the induction and the resolution of inflammation [56,57]. Human dermal macrophages express high levels of IL-36R, and IL-36 cytokines act particularly on the anti-inflammatory M2 macrophages, driving them to a pro-inflammatory M1 phenotype [53,54] (Table 1). Interestingly, Wittmann’s group has recently demonstrated that IL-36R activation by IL-36γ induces the production of psoriasis-associated cytokines (i.e., IL-23 and TNF-α) from human macrophages, and that this response is enhanced in macrophages from psoriasis patients [23]. This effect is specific for IL-36γ and could not be mimicked by other IL-1 family members, such as IL-1α [23]. Furthermore, IL-36γ-stimulated psoriatic macrophages potently activated endothelial cells and led to increased adherence of monocytes, which release IL-23, thus promoting polarization of lymphocytes with an increased IL-17/IL-22 expression [23]. Finally, neutrophils do not express IL-36R complex (Table 1). However, IL-36s indirectly contributes to their recruitment and activation in psoriatic skin, by inducing the release of the neutrophil chemoattractant molecules, such as CXCL8, CXCL1, CCL20, and LCN2, by epidermal keratinocytes [13,50].

As a whole, IL-36R activation may have a central and pivotal role in both early and chronic phases of psoriasis development, by acting at the interface of innate and adaptive immunity.

## 5. Impact of IL-36R Manipulation in Preclinical Models of Skin Inflammation

The impact of the manipulation of IL-36 axis has been widely investigated in several experimental models of psoriasis. Mice deficient of IL-36R or treated with IL-36R blocking antibodies are protected from skin inflammation induced by IMQ, an activator of TLR7 able to induce the pro-inflammatory IL-23/IL-17 axis [58,59]. Similarly, a xenograft model of psoriasis treated with an anti-human IL-36R antibody showed a significant reduction of epidermal thickness and inflammatory immune infiltrate [30]. IL-36R antagonistic antibodies inhibit inflammatory responses also in other preclinical models of psoriasiform dermatitis, such as in murine models of IL-23 or IL-36α ear-injection, which recapitulate some of key features of psoriatic skin lesions, including leucocyte infiltration and acanthosis. In these models, the inhibition of IL-36 pathway results in a significant attenuation of skin thickening and psoriasis-relevant gene expression [60]. On the other hand, IL-36Ra antagonist–deficient mice show exacerbated IMQ-induced psoriasiform phenotype [59]. Among IL-36 agonists, IL-36α is essential for the development of murine psoriasis [61]. In fact, transgenic mice overexpressing IL-36α in keratinocytes show a transient but severe skin inflammation at birth, which waned after 2–3 weeks of age. This inflammatory skin phenotype was aggravated by IL-36Ra deletion [62]. *Vice versa*, IL-36α deletion results in the amelioration of IMQ-induced skin lesions, with the reduction of epidermal neutrophils and keratinocyte acanthosis, while the specific deficiency of IL-36β or IL-36γ does not affect the disease severity [63].

Overall, IL-36 receptor inhibition could represent a promising therapeutic strategy for treating psoriasis, especially the pustular forms, in which the hyperactivation of IL-36 axis contributes to the repeated recruitment and activation of neutrophils.

## 6. Therapeutic Potential of IL36R-Targeting in Psoriasis

Psoriasis is a complex and heterogeneous inflammatory skin disease, which can manifest as different clinical forms, characterized by distinct genetic backgrounds and inflammatory signatures [64]. Pustular psoriasis, including GPP and PPP, is commonly characterized by innate immune responses typical of acute skin lesions of plaque psoriasis, where IL-1/IL-36 inflammatory axis plays a pathogenic role in the recruitment and activation of neutrophils [24]. In contrast, chronic plaque psoriasis is mainly characterized by adaptive Th17/Th1 immune responses, in which the inflammatory cytokines IL-17A and IFN-γ predominate.

Several studies reported that serum and skin levels of IL-36γ are elevated in patients with psoriasis and are closely associated with disease activity [13,16,65]. Concomitantly to the increased levels of IL-36γ, IL-38 levels strongly drop in patients affected by plaque psoriasis, thus altering the pro-inflammatory/anti-inflammatory balance of IL-36 [13]. To date, data on IL-38 expression in other clinical subtypes, such as GPP and PPP, are lacking. Importantly, the significance of the pathogenic role of IL-36 in psoriasis is supported by the observation that the therapeutic efficacy of topical or systemic drugs used for psoriasis treatment positively correlates with a reduction of circulating and skin levels of the pro-inflammatory IL-36 agonists [16,66] and an increase of the anti-inflammatory IL-38 antagonist [13].

These findings, together with the genetic studies in psoriatic patients, encouraged the employment of IL-36R blocking antibodies in clinical trials. Currently, ANB019 is under phase II study for GPP and PPP treatment. As additional strategies to suppress IL-36 activity, small molecules inhibiting the elastases responsible for the maturation of IL-36 agonists have been recently developed and tested in preclinical models of psoriasis [67].

Due to its anti-inflammatory and anti-angiogenic properties, the therapeutic use of IL-38 cytokine might represent an alternative approach to block IL-36R activation in psoriasis and in other IL-17/IL-36-dependent inflammatory diseases. In support of this hypothesis, the administration of a recombinant full-length form of IL-38 attenuates the severity of the disease in the IMQ-induced murine model of psoriasis, by reducing the local inflammation and restoring the epidermal differentiation [13]. Consistently, a mature variant of IL-38 also ameliorated the psoriasiform phenotype induced by IMQ and this effect was more pronounced in IL38 KO^-/-^ mice compared to wild-type counterparts. These data suggest that a potential therapy with exogenous IL-38 could be addressed to the inflammatory diseases characterized by low levels of endogenous IL-38, such as plaque psoriasis [38]. Interestingly, IL-38 has the peculiarity to target more than one receptor of the IL-1 family and to interfere with more than one cytokine, including IL-17, IL-36, and TNF-α [31]. IL-38 is also able to inhibit TLR4 signaling and to reduce indirectly the release of IL-36γ, as well as the production of IL-6 and IL-23 by human macrophages [68].

Due to the broad range of receptors, potentially targeted by IL-38 and differently activated during the course of psoriasis, IL-38 manipulation could represent a valid therapeutic strategy for the treatment of acute and chronic psoriatic skin lesions, as well as of other disease subtypes. Importantly, this peculiarity of IL-38 could circumvent inherent drawbacks of the current biological drugs, targeting TNF-α or IL-17 cytokines, such as immune mechanisms of redundancy and compensation, which can lead to therapy resistance or adverse reactions, as described for anti-TNF-α or anti-IL-17A drugs [69].

## 7. Conclusions

In this review, we focused on the pathogenic role of IL-36R activation in psoriasis, with an emphasis on its effects on the inflammatory responses evoked by resident skin cells and immune cells during psoriasis development. In addition to IL-36R blocking by neutralizing antibodies, the reinforcement of IL-38 could represent a prosecutable therapeutic strategy for inhibiting IL-36-induced responses in psoriasis. IL-38 augmentation strategy could be effective also for other inflammatory skin diseases with an abundant neutrophil infiltrate such as GPP, PPP, hidradenitis suppurativa, Sweet syndrome and pyoderma gangrenosum, in which a reduced expression of IL-38 has been observed. However, a more detailed understanding of the functional biology of IL-38 in skin context to foresee the potential impact of its manipulation in psoriasis treatment is needed. In particular, a better molecular and functional characterization of IL-1RAPL1 receptor, a newly identified inflammatory target of IL-38, in other immune and resident skin cells, might unveil novel IL-23/IL-17-dependent pathways potentially controlled by IL-38.

## Figures and Tables

**Table 1:** Expression of IL-36 members and IL-36R complex in skin resident and immune cells present in psoriatic skin lesions.

IL-36 Members	Expression	Induction	Dow-Regulation	Refs
IL-36α (IL-1F6)	Keratinocytes, endothelial cells, macrophages, dendritic cells, Langerhans cells	IL-17A, TNF-α, IL-22		[13,17,18,49]
IL-36β (IL-1F8)	Endothelial cells	IL-36β, IL-17A, TNF-α, IL-22, IL-1β, LPS		[13,17,18,49]
IL-36γ (IL-1F9)	Keratinocytes, endothelial cells, macrophages, dendritic cells, Langerhans cells	IL-17A, TNF-α, IL-36γ, TLR3, LPS, NETs		[13,17,18,49,50]
IL-36Ra (IL-1F10)	Keratinocytes	IL-17A, TNF-α, IL.36γ		[13,17,18,49]
IL-38 (IL-1F5)	Keratinocytes		IL-17A, TNF-α IL-22, IL-36γ, IFN-γ	[13,34]
IL-36R complex	Keratinocyte, fibroblasts, endothelial cells, macrophages, dendritic cells, Langerhans cells			[13,14,19,21,23,53,54,55]

## Abbreviations

IL-1R	IL-1 Receptor
IL-1Ra	IL-1 Receptor antagonist
IL1RAPL1	X-linked interleukin-1 receptor accessory protein-like 1
JNK	Jun N-terminal Kinase
MyD88	Myeloid Differentiation Primary Response Protein 88
MAPK	Mitogen-Activated Protein Kinases
IkBz	Nuclear Factor-kappa B inhibitor zeta
NFkB	Nuclear Factor-kappa B
TRAF	TNF Receptor-Associated Factor
IRAK	Interleukin 1 Receptor Associated Kinase 1
STAT	Signal Transducer and Activator of Transcription
